# Does video feedback analysis improve CPR performance in phase 5 medical students?

**DOI:** 10.1186/s12909-016-0726-x

**Published:** 2016-08-12

**Authors:** Andrew D. Spence, Sonia Derbyshire, Ian K. Walsh, James M. Murray

**Affiliations:** The Clinical Skills Education Centre, Medical Biology Centre, Queen’s University Belfast, 97 Lisburn Road, Belfast, BT9 7BL Northern Ireland UK

**Keywords:** Undergraduate medical education, Cardiopulmonary resuscitation, Curriculum development, Video technology, Feedback

## Abstract

**Background:**

The use of simulation in medical education is increasing, with students taught and assessed using simulated patients and manikins. Medical students at Queen’s University of Belfast are taught advanced life support cardiopulmonary resuscitation as part of the undergraduate curriculum. Teaching and feedback in these skills have been developed in Queen’s University with high-fidelity manikins. This study aimed to evaluate the effectiveness of video compared to verbal feedback in assessment of student cardiopulmonary resuscitation performance.

**Methods:**

Final year students participated in this study using a high-fidelity manikin, in the Clinical Skills Centre, Queen’s University Belfast. Cohort A received verbal feedback only on their performance and cohort B received video feedback only. Video analysis using ‘StudioCode’ software was distributed to students. Each group returned for a second scenario and evaluation 4 weeks later. An assessment tool was created for performance assessment, which included individual skill and global score evaluation.

**Results:**

One hundred thirty eight final year medical students completed the study. 62 % were female and the mean age was 23.9 years. Students having video feedback had significantly greater improvement in overall scores compared to those receiving verbal feedback (*p* = 0.006, 95 % CI: 2.8–15.8). Individual skills, including ventilation quality and global score were significantly better with video feedback (*p* = 0.002 and *p* < 0.001, respectively) when compared with cohort A. There was a positive change in overall score for cohort B from session one to session two (*p* < 0.001, 95 % CI: 6.3–15.8) indicating video feedback significantly benefited skill retention. In addition, using video feedback showed a significant improvement in the global score (*p* < 0.001, 95 % CI: 3.3–7.2) and drug administration timing (*p* = 0.004, 95 % CI: 0.7–3.8) of cohort B participants, from session one to session two.

**Conclusions:**

There is increased use of simulation in medicine but a paucity of published data comparing feedback methods in cardiopulmonary resuscitation training. Our study shows the use of video feedback when teaching cardiopulmonary resuscitation is more effective than verbal feedback, and enhances skill retention. This is one of the first studies to demonstrate the benefit of video feedback in cardiopulmonary resuscitation teaching.

**Electronic supplementary material:**

The online version of this article (doi:10.1186/s12909-016-0726-x) contains supplementary material, which is available to authorized users.

## Background

Simulation within medical education is increasing, with undergraduate students clinically taught and assessed using simulated patients and manikins [1]. There have been developments in manikin technology and medical education facilitating different interactive feedback methodologies and advanced debriefing techniques [1, 2]. Allied to this technology, performance analysis using video feedback is increasingly utilised in medical education in our courses [3]. Mainly used in communication skills training, video feedback has been shown to be beneficial in enhancing medical student education [4]. Cardiopulmonary resuscitation (CPR) training in the Queen’s University Belfast (QUB) undergraduate medical curriculum is an essential component to prepare students for postgraduate work. Post-scenario feedback has traditionally been verbal, however, Yeung has shown there is strong evidence for the use of technology for feedback in CPR training, using simulation-based medicine [3].

As correctly performed chest compressions produce 17–27 % of normal cardiac output, effective CPR training sessions in undergraduate medicine is of paramount importance [5]. The primary outcome of this study was to analyse the overall effectiveness of video compared with verbal feedback in teaching phase five (final year) medical students CPR. Secondary outcomes included analyses of auscultation, compression and ventilation quality comparison between the two cohorts (video and verbal feedback). A checklist assessment tool was developed for use in this study to increase objectivity in scoring of students following the CPR algorithm [6].

## Methods

### Aims

The primary aim of this study was to determine if video feedback in a CPR scenario is superior to verbal feedback. The secondary aim was to assess the individual abilities used during CPR to determine the effectiveness of video feedback on skill performance.

### Characteristics of participants

**Fig. 1:**
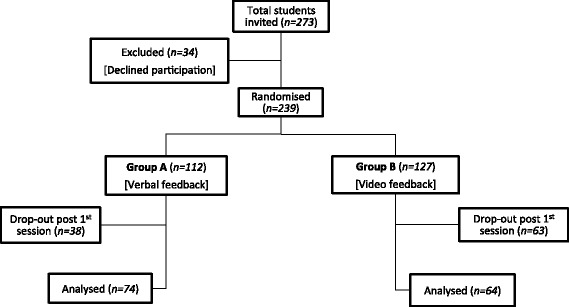
Study flow chart

Following University Research Ethics Committee approval, all 273 students in final year medicine (Phase 5) at the Queen’s University of Belfast were invited to participate in the study. Final year students who consented to participate were allocated to one of two cohorts, using Microsoft Excel**®** to pseudo-randomise them via anonymous student university identification numbers, resulting in one cohort receiving verbal feedback (cohort ‘A’) and a second cohort receiving video feedback (cohort ‘B’). The study flow chart is displayed in Fig. 1. Each student had the same experience of ALS prior to the study, receiving one session of ALS training of similar scenarios in the previous academic year (4^th^ year Medicine in Queen’s University Belfast).

### Design & setting

All participants undertook a pre-determined CPR scenario (*in hospital cardiac arrest with ventricular fibrillation*) using a Laerdal**®** Resusci-Anne QCPR adult training manikin (Laerdal Medical Corporation, Stavanger, Norway). The students were assigned to teams of five for the scenario. As the total number of students that consented to participate was not divisible by five, teams of four were used to ensure students who consented were not excluded, resulting in a similar number of groups of four and five between cohorts, limiting this as a form of bias. Each team performed the same scenario from discovery of the unconscious patient to termination – when the patient regained consciousness. Consistency was maintained by standardising the scenario for all groups. The cohorts were alternated on a weekly basis so the remaining groups of one cohort would not have a substantial advantage over the other in order to reduce this potential bias. The scenario, ventricular fibrillation, was the same for each group as variation to another, such as pulseless electrical activity, may have influenced student performance. Furthermore, if pulseless electrical activity were used as an alternative to the ventricular fibrillation scenario then defibrillation would not be performed, introducing inconsistency in assessment between groups that undertook different scenarios.

Verbal feedback was given to each group of students in cohort A immediately post-scenario, over a 10-min period by two independent assessors, who were trained in assessing and providing feedback in clinical scenarios undertaken by students, and who had scored the students during the scenario. The verbal feedback was based on the areas performed poorly and well according to the scoring on the structured checklist assessment tool, maintaining consistency as the video feedback was also based on the scoring using this tool. The students in the videoed cohort received an analysed video of their performance by an online QUB Dropbox**™** account, but were not given verbal feedback.

Data were collected using video recording equipment. The two independent assessors used the checklist assessment tool to score each team with respect to individual variables, global score of performance (maximum 10 points) and total score (maximum 90 points) (Additional file 1: Figure S1). As each student would not be performing each skill during the scenario the students were scored as a team instead of individually. Each scenario lasted for approximately 10 min at which point it was halted. This time frame ensured each group should be able reach the point in the scenario when they have delivered three shocks to the simulated patient. This allowed for assessment of the students’ knowledge of drug delivery after the 3^rd^ shock, as stated in the ALS algorithm [6]. Feedback for both cohorts, one verbally and one via video, was provided based on marks awarded on the checklist assessment tool, highlighting areas performed positively and poorly, with appropriate constructive criticism. The videos from cohort B were analysed by one researcher using a commercial video analysis software program – StudioCode^**©**^. Studiocode is a video analysis tool designed for use within healthcare, education, and research sectors to measure, analyse, and improve performance. It can be used to objectively score a student or trainee at a set task. In this study, prior to the scenario, a scoring system was coded that was used for assessment. The scenario was divided into separate components that were scored individually and then totalled, producing an overall numerical score. As the assessor scored the video performance, Studiocode recorded, labelled, and applied the score to this section. This allowed targeted annotated feedback for each skill component. Videos, complete with scoring and targeted component feedback, were then available to each student for personal review. The feedback in video clips were limited to those skills assessed using the checklist in order to avoid providing additional feedback not explored in the sessions with cohort A.

The videos were distributed to students via a password protected QUB Dropbox**™** account. They were only able to view their own video and were not able to access other groups’ videos. There were no issues reported regarding access to the videos, and students were already familiar with this method of resource delivery through other courses in the curriculum utilising Dropbox**™**. Due to the time taken to analyse, refine annotation and distribute the videos each one was made available within one week of the scenario. The only cost in the study was a StudioCode licence fee, which had already been obtained by QUB for use in other projects.

Each student was invited to return 4 weeks post-first session for a second scenario. To maintain consistency the checklist assessment tool and analysis of the return scenario was identical to the first. To minimise disruption to the students’ clinical placements the sessions were held on 1 day per week, on each Tuesday composing of either cohort A or cohort B. These logistical reasons resulted in the 9 week duration of the study. On return the students were crossed over, when cohort A received video feedback and cohort B received immediate verbal feedback. This was conducted using the same methods as used in the first session. The scenarios took place in a specialised clinical skills room that contained two overhead cameras for video recording. These were linked to computer software in an adjacent room. The video recording commenced when the student entered the room. The recording ceased after the students had been given the opportunity to achieve all points on the mark sheet – i.e. when the ‘patient’ showed signs of life. This occurred after administration of the drugs (i.e. after the third defibrillation). The scenario was also videoed by a fellow researcher using an iPad**™**, the purpose of which ensured if the overhead cameras were obscured by students, thus impacting on assessment and video feedback, the iPad**™** footage could be incorporated into the StudioCode feedback clips. However, this iPad**™** video recording was not required as a clear view was maintained using the ceiling cameras alone.

### Statistical analysis

We defined the main outcome variable as change in score immediately post-test and the true standard deviation associated with this measure represented by *s*. If the true difference in change of score between verbal and video feedback exceeded 0.5*s, then a study with 64 participants per cohort would have in excess of 80 % power to detect this difference, assuming a two-sided hypothesis and a significance level of 5 %. As the scores obtained by the participants was a continuous variable independent student’s t tests were used to determine statistical significance. The variable of interest was the differential change in score (total, global and individual skill variables) under verbal and video feedback with the standard deviation defined in similar terms. A General Linear Model with interactions was used to examine the differential effect of video feedback against verbal feedback over time. The total score statistics were analysed using this general linear model, whereas the individual variables and global scores were analysed using ordinal regression. Analysis was conducted using SPSS^**©**^ version 21 statistical software (SPSS inc. Chicago, Il, USA).

## Results

### Participants

The total number of students invited to participate in the study was 273 and the number of consenting students was 239. One hundred and forty seven (62 %) participants were female. The mean age was 23.9 years (range 22.1–46.7 years). The number of students who completed the entire study was 138. This large dropout rate was due to the study timing in the academic year - prior to the students’ written final exams. During study design the authors considered this limitation, however due to the structure of the medical curriculum we were only able to conduct our study during the autumn semester of final year, a few weeks prior to the exams.

### Feedback scoring

The average improvement in total score was greater in cohort B (12/90 points) when compared to cohort A (4/90 points). Twenty-one of the groups in cohort B improved score compared with 14 groups of cohort A. Nine groups in cohort A achieved lower scores in the return scenario, compared with three groups in cohort B (Table 1).

**Table 1:** Comparison of verbal feedback against video feedback in a CPR scenario

Variable	Cohort A	Cohort B
Mean improvement in total score^a^	4	12
Range of total scores in the return session^a^	68–84	65–88
Largest increase in total score for a single group^a^	30	47
Number of groups improving overall score between sessions	14	21
Number of groups achieving a lower score at the return session	9	3

^a^Maximum overall score = 90

Table 2 shows the significantly greater increase in total score for cohort B when compared with cohort A (*p* = 0.006, 95 % CI: 2.8–15.8). When the individual skills within the scenario are analysed there is a greater improvement in performance in cohort B compared with cohort A from first session to return session for ventilation quality and global score (*p* = 0.002 and <0.001, respectively). Despite drug administration timing not showing a statistically significant improvement at the 5 % level, it was significant at the 10 % level (*p* = 0.059). Although there was, on average, a greater increase in scores for the other skill variables, shown in the checklist tool, this difference was not statistically significant.

**Table 2:** Change in scores for verbal feedback and video feedback from session one to session two

Variable	Mean change in score		Cohort B more effective than cohort A		Improvement in cohort B scores between sessions	
	Cohort A	Cohort B	*p* value	95 % CI^a^	*p* value	95 % CI^a^
Total score^b^	4	12	.006	2.8, 15.8	< .001	6.3, 15.8
Global score^c^	1.8	2.5	< .001	1.8, 6.0	< .001	3.3, 7.2
Auscultation^d^	0.8	2.0	.087	−0.2, 3.6	.384	−0.7, 1.9
Compression depth^d^	0.8	0.3	.312	−1.1, 3.3	§	§
iVentilation quality^d^	−1.0	0.3	.002	1.2, 5.0	.124	−0.3, 2.3
Drug administration timing^d^	0.6	1.8	.059	−0.7, 4.1	.004	0.7, 4.1

^a^CI = Confidence intervals; ^b^Maximum score = 90; ^c^Maximum score = 10; ^d^Maximum score = 4

§ Analysis not possible due to the ordinal model on compression depth failing to provide reliable estimates - no student pre-feedback attains a score of 2 and no student post-feedback attains a score of 0, creating issues with the modelling process

Data presented in Table 2 show a positive change in total score for cohort B from session one to session two (*p* < 0.001, 95 % CI: 6.3–15.8). In addition, using video feedback showed a significant improvement in the global score of participants (*p* < 0.001, 95 % CI: 3.3–7.2) and drug administration timing (*p* = 0.004, 95 % CI: 0.7–3.8). Although other variables, on average, improved scores from the first session to the return session in cohort B these increases in scores did not reach statistical significance.

Skill variables were analysed to determine if there was a statistically significant increase in score achieved in the return session when the verbal feedback cohort (A) was compared with the cohort receiving initial video feedback (B). This allows comparison of cohort A against cohort B in terms of preparation for the scenario, without considering the scores from the first session. Variables ventilation quality (*p* = 0.06) and remembering to auscultate (*p* = 0.028) demonstrated viewing the video of the scenario was significantly superior to preparation after receiving verbal feedback. However, the total score was unaffected between cohorts (*p* = 0.194).

## Discussion

This study shows there is a significant advantage in the effectiveness of feedback using video when compared to a verbal method in the overall teaching of CPR in a simulation environment (*p* = 0.006). The study also demonstrates there are specific individual skills within the ALS algorithm that are enhanced by video feedback, compared to verbal feedback (ventilation quality [*p* = 0.002] and global score [*p* < 0.001]). The other variables in the checklist assessment tool were not affected by the use of video compared to verbal feedback. However, despite not achieving statistical significance (at the 5 % level), variables such as drug administration timing showed video feedback had a positive trend on skill retention from the first to return session (*p* = 0.059). Our study also demonstrated that using video feedback in CPR training improves global scores (*p* < 0.001). Combining this with improvement of overall scores confirms previous evidence that high-fidelity simulation has a statistically significant correlation between checklist and global scores [7].

Applying modern feedback technology to real-life clinical situations, Kramer-Johansen *et al.* demonstrated its use in CPR training is associated with improved short-term patient survival [8]. Our study has shown video feedback technology improves not only overall performance but also individual skill (ventilation) in a simulation setting. Further research of our feedback methods is required to determine the effectiveness in a real-life clinical scenario and impact on patient survival. Our video feedback methodology provided a mechanism for students to view their own, and their colleagues’ performances. They were able to gain feedback from a variety of modalities (chest compressions, ventilation, defibrillation, drug administration) even though they had not performed all of these skills personally during the scenario. Although the greater marks in quality of chest compressions was not statistically significant there was a trend toward an improvement in scores in the video feedback cohort. Observations included students’ adopting correct hand position during chest compressions for the return scenario. This feedback was relayed using annotated video clips on the software. Correct hand position has been proven to produce a greater cardiac output during simulation and thus utilising video feedback using methodology, such as in our study, has the potential to improve performance [5].

Decay of skills acquired using simulation is another important factor to be considered. Barrett has shown the value of repetition in skill acquisition and retention thus reducing the rate of skill loss [9]. Although skill retention was improved in our video feedback participants, in the four-week period, decay of CPR technique and knowledge acquired using this method should be investigated further to determine its long-term effectiveness. An advantage of annotated video feedback is the student can review it as many times as they wish, improving retention of skill, as shown in this study. Our findings mirror a study of radiology teaching by Corr, who showed a major advantage of online learning is content can be downloaded and viewed multiple times, thereby enhancing reinforcement of knowledge and skill [10].

As discussed, there was a large dropout rate in student participation, a limitation of the study. This was due to the timing of the return component of the study being a few weeks prior to the written final exams. There was a greater dropout rate in cohort B due to this cohort’s return sessions being scheduled closest to the exams. Despite this limitation the study still maintained a sufficient number of participants to obtain 80 % power and to produce significant results. Also, to include all students who consented we created groups of four, as opposed to excluding some students due to the total not being divisible into equal groups of five. This was a limitation to the methods, however when scores were compared it did not alter the results. To maintain consistency with the verbal feedback cohort the video feedback clips also totalled 10 min. As the students were able to download their Dropbox**™** videos to their personal computer we could not monitor the number of times each student viewed their video. On questioning during the return session the students all reported they downloaded and watched their video, however they unable to reliably report the exact number of times they watched their video.

An additional limitation was the students receiving video feedback one week post-scenario, compared to cohort A who received their verbal feedback immediately after the scenario. Due to the laborious nature of analysing, annotating and distributing the video files to the students this limitation could only have been reduced by using more than one assessor for this methodology. The resultant delay leaves the potential for self-reflection during this period, possibly enhancing learning, in addition to the subsequent video review. A solution of having more than one assessor to analyse the videos would have potentially reduced consistency in scoring of skill and so also has drawbacks.

We recognise the study methodology is prone to bias where we delivered verbal feedback to a group of students whereas each student downloaded their video for viewing, which most likely happened alone rather than in groups. This could lead to students benefiting more from one type of feedback if they are learners who gain more from being in groups or by learning alone. It was not feasible to arrange the students to watch their videos together due to the course hospital placements being province-wide. A solution would be to provide verbal feedback to each student individually, however this would require five assessors to deliver feedback after each scenario, which itself would introduce bias. A confounding factor for the improvement in scoring for cohort B may be the initial marks could have been an extreme low and, although video feedback may have aided in the improvement, a degree of return to average skill performance could have contributed to the increased scores. To assess this possibility ideally we would have repeated the scenario a third time and assessed student performance again. The hypothesis being cohort A (receiving video feedback in the second scenario) would demonstrate a significantly greater improvement in skill when compared to cohort B (receiving verbal feedback in the second scenario), between the second and third sessions. Unfortunately this was not possible due to the curriculum design and a clash with exams. We suggest future research using cross-over study design for video feedback assessment should include further follow-up scenarios to enhance the reliability of the results.

As an additional use we propose video recording technology could be utilised to provide a record of achieved competency in particular skill areas. This objective documentation in video format could lead to a student ‘passport’ of skills and achieved competencies, which would be helpful to students, their teachers and future employers. This would be part of the students’ portfolios and later appraisal folders. This study shows the use of video technology as a feedback method is effective in a simulation teaching environment. However, further research is required to determine if video feedback, using similar methodology to our study, improves CPR skill retention on a longer-term basis, in addition to investigating ‘transfer to practice’ as there is a lack of data studying the transference of skill from simulation training at undergraduate level to the workplace [11].

## Conclusions

Video feedback technology is a rapidly developing area of simulation-based medicine. We have shown its effectiveness in retaining and improving students’ skills in CPR, both in overall performance and on an individual skill level providing an objective demonstration of student ability. However, there are limitations to assessment using this technology and as such further research using video feedback in cross-over studies should be undertaken.

## Additional file

Additional file 1: Figure S1. Checklist assessment tool for scoring student performance. (PDF 188 kb)

## Abbreviations

ALS: Advanced Life Support
CPR: Cardiopulmonary Resuscitation
PEA: Pulseless Electrical Activity
QUB: Queen’s University Belfast
