# Supplementary material for: Does video feedback analysis improve CPR performance in phase 5 medical students?
Source: BMC Med Educ. 2016 Aug 12;16:203. doi: 10.1186/s12909-016-0726-x (PMC4983021; doi:10.1186/s12909-016-0726-x)
Supplement: Additional file 1: Figure S1. — Checklist assessment tool for scoring student performance. (PDF 188 kb) [file 12909_2016_726_MOESM1_ESM.pdf]

Queen's University Belfast

Researcher marking sheet – (non studiocode) Ref: 12.39

'The Comparison of CPR Performance in Phase 5 Medical Students with and without the use of video feedback analysis'

Date and time .... Initials and signature of researcher ....

Group number:

Student action						***Score***
Check responsiveness			Done (4)		Not done (0)	
Call resuscitation team			Done (4)		Not done (0)	
Attach monitor/defibrillator/pads			Done (4)		Not done (0)	
Airway	Bag/Mask airway		Done (4)		Not done (0)	
	Bag/Mask airway	Chest rise	Full (4)	Partial (2)	None (0)	
	Supraglottic airway	Chest rise	Full (4)	Partial (2)	None (0)	
	Supraglottic airway		Done (4)		Not done (0)	
	Auscultates chest		Done (4)		Not done (0)	
Rhythm recognition (VF)			Correct (4)		Incorrect (0)	
Rate of compressions			Good [>100] (4)		Poor [<100] (0)	
Depth of compressions			Good (4)	Average (2)	Poor (0)	
ALS algorithm sequence followed			Yes (4)		No (0)	
Defibrillation	*Energy*		Correct (4)		Incorrect (0)	
	Pad placement		Correct (4)		Incorrect (0)	
	Safe defibrillation		Yes (4)		No (0)	
Vascular access			Achieved (4)		Not achieved (0)	
Drug administration timing			Correct (4)		Incorrect (0)	
Correct drug choice e.g. epinephrine			Correct (4)		Incorrect (0)	
Correct dose of drug administered			Correct (4)		Incorrect (0)	
CPR cessation timing (signs of life)			Appropriate (4)		Inappropriate (0)	
Total Score						/80
Facilitator global score			Excellent (10)			
			Good (6)			
			Borderline (2)			
			Fail (0)			
Overall Total Score						/90
